# Nanoporous Amorphous Carbon Monolayer Derived from Fullerene Film

**DOI:** 10.1002/advs.202308187

**Published:** 2023-12-28

**Authors:** Meng He, Yehui Ding, Xue Liu

**Affiliations:** ^1^ College of New Energy Xi'an Shiyou University Xi'an 710065 China; ^2^ State Key Laboratory for Mechanical Behaviour of Materials Xi'an Jiaotong University Xi'an 710049 China

**Keywords:** amorphous carbon monolayer, fullerene, nanoporous

## Abstract

Carbon materials derived from fullerene are reported recently with unique structures and properties. However, only micrometer size samples can be obtained that limits the studying and exploration for membrane applications. Here, the preparation of a centimeter‐size nanoporous amorphous carbon monolayer is reported by rapid pyrolyzing a Langmuir–Blodgett film of fullerene. The sample is fully characterized and the results indicate that the amorphous carbon monolayer derived from fullerene is metastable and insulating. The ionic transmembrane transport study demonstrates that the membrane is porous and cation selective with a selectivity of 26%. This work provides new insights into the controlled synthesis of large‐size metastable amorphous carbon monolayer.

## Graphical abstract

A large‐size amorphous carbon monolayer is fabricated by rapid pyrolyzing the fullerene monolayer. The amorphous carbon monolayer is metastable, insulating, and nanoporous with a cation selectivity of 26%. This work provides new insight into the controlled synthesis of large‐size metastable amorphous carbon monolayer.

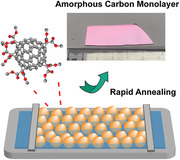

## 1 Introduction

Ultrathin nanoporous membrane has great potential for a wide range of applications, for instance, desalination,^[^ ^1^ ^]^ blue energy generation,^[^ ^2^ ^]^ and biomedical applications.^[^ ^3^ ^]^ The ultrathin nature offers low transmembrane transport resistance and large permeability.^[^ ^4^ ^]^ Among them, nanoporous carbon membrane has particularly drawn intense attention due to their outstanding chemical and mechanical stability. The nanopores can be introduced into the pristine graphene monolayer by O_2_ plasma, ion bombardment, and electron beams.^[^ ^5^ ^]^ However, the main hurdle is the complicated operation and expensive setup. As a comparison, a centimeter size nanoporous amorphous carbon monolayer can be prepared by electron irradiation^[^ ^6^ ^]^ and thermal annealing of precursor molecules,^[^ ^7^ ^]^ which exhibits great potential for fundamental research and technical applications.^[^ ^8^ ^]^ The preparation of an amorphous carbon monolayer usually requires a high concentration of active carbon species which provides high nucleation density, and the main challenge is to prevent multilayer growth while providing a large amount of carbon precursors.^[^ ^9^ ^]^ Besides, the precursors are usually polyaromatic hydrocarbons (PAHs) with planar structures. The curved PAHs like fullerene, however, have not yet been used for amorphous carbon monolayer preparation, the properties of which have not been studied. Recent experimental and theoretical studies show that amorphous carbon monolayer has distinct electronic properties.^[^ ^7^, ^9^, ^10^ ^]^ For instance, the sheet resistance of amorphous carbon monolayer can be increased by up to 10^9^ times with a 25‐degree rise in preparation temperature.^[^ ^7c^ ^]^ Considering the potential applications of amorphous carbon monolayer for nanoelectronics, the preparation of amorphous carbon monolayer directly on Si wafers is of value which can avoid the damage and polymer residues associated with the transfer process.

Recently, new types of carbon monolayers have been fabricated by using fullerene as precursors.^[^ ^11^ ^]^ Among them, an amorphous carbon monolayer has been prepared by pyrolyzing fullerene at ambient pressure, which is produced during the transformation from fullerene‐type to graphene‐type carbons.^[^ ^11c^ ^]^ It is metastable and turned into graphene‐type carbon by annealing above 600 °C. The HRTEM images show that the metastable structure contains interesting nanopores formed between the crosslinked broken fullerene cages. However, limited by the size of the sample, it cannot be exfoliated into a monolayer that is large enough for a nanopore properties study. The direct preparation of a centimeter‐sized amorphous carbon monolayer from fullerene is clearly helpful in this case.

We reported previously the preparation of amorphous carbon monolayer by the rapid thermal processing (RTP) treatment of Langmuir–Blodgett film of planar PAHs, and the membrane properties are influenced by the PAHs structure.^[^ ^7a^ ^]^ Herein, we report the fabrication of a nanoporous amorphous carbon monolayer from the Langmuir–Blodgett film of fullerene. We found that the amorphous carbon monolayer thus prepared is metastable and has lower thermal stability than that prepared with planar PAHs. The amorphous carbon membrane is electrically insulating and the ion transmembrane transport studies show that the amorphous carbon monolayer is cation‐selective with a selectivity of 26%, making it a promising material for nanoelectronics and membrane applications.

## 2 Results and Discussion

**Figure 1:**
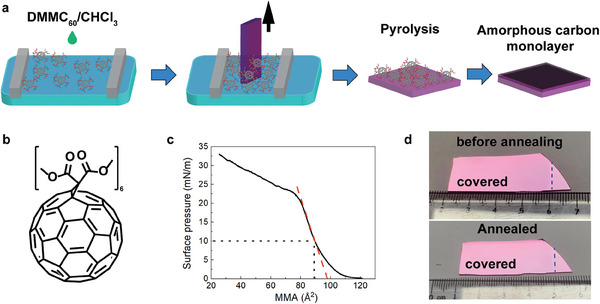
The illustration of amorphous carbon monolayer preparation from DMMC_60_ film. a) Scheme of amorphous carbon monolayer preparation. b) The chemical structure of DMMC_60_. c) The Langmuir compress isotherm of DMMC_60_ on the water subphase. The mean molecular area is ≈97 Å^2^, close to the DMMC_60_ molecule size. d) Optical photos of DMMC_60_ monolayer before annealing and amorphous carbon monolayer. The dashed line indicates the edge of the monolayers. The size of the monolayer is ≈4 × 1.5 cm.

The amorphous carbon monolayer was prepared by rapid pyrolyzing the Langmuir–Blodgett film of dimethyl malonate decorated fullerene (DMMC_60_) (**Figure** **1**a). Unmodified fullerene tends to form clusters on the water surface and the uneven film was formed when transferred to substrate.^[^ ^12^ ^]^ The dimethyl malonate groups can interact with the water subphase that makes the DMMC_60_ amphiphilic and prevents the formation of fullerene clusters (Figure 1b). First, a solution of DMMC_60_ in chloroform with a concentration of 1 mg mL^−1^ was deposited at the air‐water interface in a Langmuir trough. The barrier was pushing slowly the DMMC_60_ molecules together at a speed of 1 mm min^−1^ until the surface pressure of 10 mN m^−1^ was reached. The mean molecular area (MMA) calculated from the compress isotherm is about 97 Å^2^, which is close to the projected area of DMMC_60_ (11 Å in diameter), indicating that the fullerene monolayer was formed on the surface of the water (Figure 1c).

Next, the DMMC_60_ monolayer was deposited onto a Si wafer that was pre‐treated with air plasma (150 W, 1 min) to form a hydrophilic surface. The wafer with DMMC_60_ monolayer was then annealed in a RTP oven (500 °C, Ar atmosphere, 1 bar) for 30 s (see Experimental Section for details). The amorphous carbon monolayer obtained has a size of 4 cm × 1.5 cm, and the monolayer size is mainly limited by the size of the LB deposition trough and RTP oven. The amorphous carbon monolayer on the Si wafer was used directly for further analysis or transferred onto substrates by PMMA transfer and HF etching. The amorphous carbon monolayer on the Si wafer is only visible at a specific angle (Figure 1d; Figures S1 and S2, Supporting Information).

**Figure 2:**
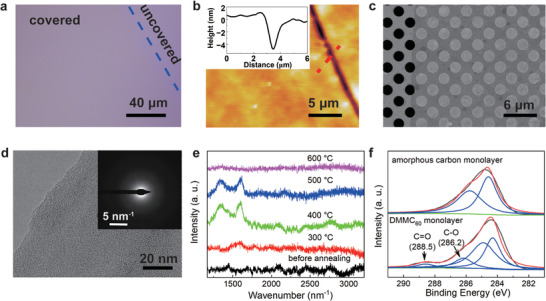
Characterization of the amorphous carbon monolayer preparation from DMMC_60_ monolayer. a) Optical image of amorphous carbon monolayer on Si wafer. b) AFM image of amorphous carbon monolayer on Si wafer, the surface roughness (Ra) is ≈220 pm. The thickness is ≈3.5 nm. c) SEM image of amorphous carbon monolayer on Quantifoil TEM grid with holes of 2 µm. d) TEM image of amorphous carbon monolayer. The SAED exhibits a characteristic diffuse halo. e) Raman spectra of amorphous carbon monolayer annealed at different temperatures. f) C refined XPS spectra of amorphous carbon monolayer and DMMC_60_ monolayer.

The amorphous carbon monolayer on the Si wafer has a lower contrast compared to the amorphous carbon membrane prepared with planar PAHs (**Figure** **2**a; Figures S3 and S4, Supporting Information).^[^ ^7^, ^8^ ^]^ The AFM images of amorphous carbon monolayer on Si wafer show a flat morphology with a surface roughness Ra of ≈220 pm (Figure 2b). The thickness of the monolayer is ≈3.5 nm, which is consistent with the monolayer nature. The SEM image of the amorphous carbon monolayer on the Quantifoil TEM grid (2 µm hole) shows that the monolayer has good mechanical strength (Figure 2c). The structure was further explored by TEM. The TEM image shows that the membrane is smooth and uniformly flat (Figure 2d). Selected area electron diffraction (SAED) pattern exhibits a characteristic diffuse halo, indicating the amorphous nature of monolayer.

The annealing temperature plays an important role in triggering the rearrangement of DMMC_60_ and the annealing process was studied by Raman spectroscopy (Figure 2e). The Raman spectrum of DMMC_60_ monolayer exhibits no fullerene Raman signals, which may due to the decomposition of DMMC_60_ during the spectrum acquisition. By increasing the annealing temperature to 400 °C, intense *D* and *G* peaks were observed, corresponding to the bond stretching of sp^3^ defects and sp^2^ pairs,^[^ ^13^ ^]^ which indicates that the crosslinking between DMMC_60_ occurred. The Raman spectra of C_60_ nanosheets and long‐range ordered porous carbon (LOPC) powder reported in the literature show typical *D* and *G* bands, which is consistent with what we observed here.^[^ ^11a,c^ ^]^ No 2D peak was observed due to the lack of long‐range order in the structure. By increasing the annealing temperature further to 600 °C, both *D* and *G* peaks were vanished, indicating the amorphous carbon monolayer was decomposed. This is consistent with the LOPC reported in literature that gradually loses the fullerene‐type metastable structure upon annealing at 600 °C.^[^ ^11c^ ^]^ The amorphous carbon monolayer prepared by using planar PAHs is stable at 900 °C in the Ar atmosphere (Figure S4, Supporting Information). The significant thermal stability difference indicates that the amorphous carbon monolayer reported here is metastable. The annealing process is also studied by XPS (Figure 2f). Before annealing, the C refined XPS spectrum of DMMC_60_ monolayer contains four peaks, corresponding to the sp^2^ carbon, sp^3^ carbon, C─O, and C═O. After annealed at 500 °C, the C peak from C─O, and C═O are fully vanished, and the spectrum is mainly dominated by sp^2^ and sp^3^ carbon, which indicates that the dimethyl malonate group were fully decomposed during the annealing process.

**Figure 3:**
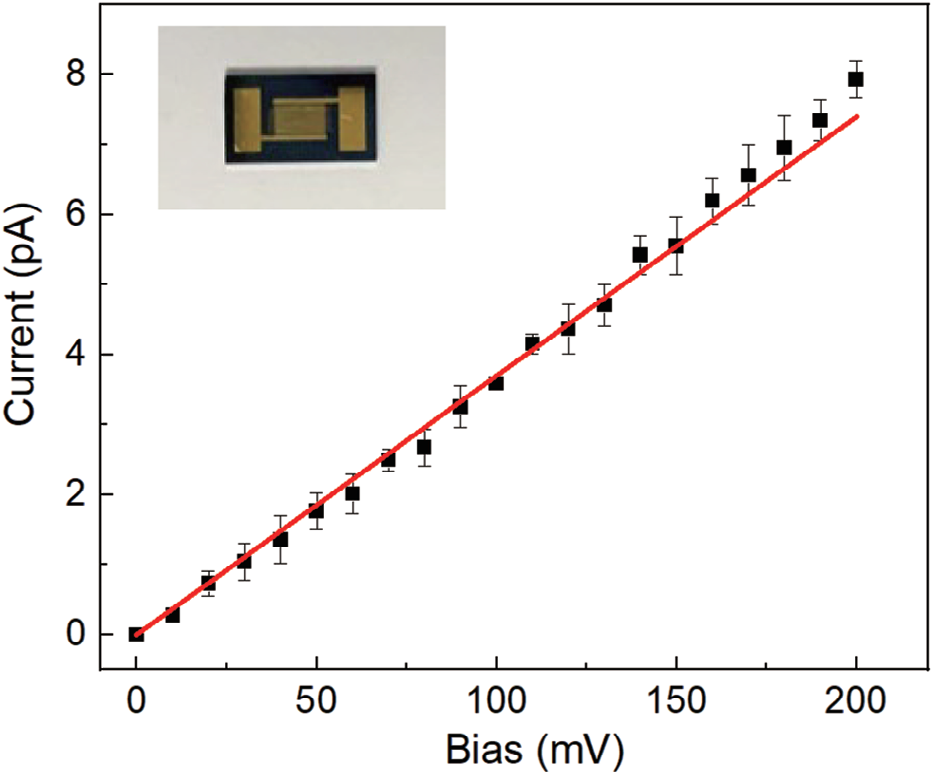
Conductivity measurement *I*–*V* plot of the amorphous carbon monolayer preparation from DMMC_60_ monolayer. insert an interdigital electrode with a sample for the conductivity measurement.

The electrical conductivity of the amorphous carbon monolayer prepared at 500 °C was studied (**Figure** **3**). The monolayer was transferred onto the interdigital electrode by using standard PMMA transfer and HF etching (see Experimental Section for details). As shown in Figure 3, the sheet resistance value of amorphous carbon monolayers generated from DMMC_60_ is ≈26.3 GΩ sq^−1^, similar to the amorphous carbon monolayer reported in the literature.^[^ ^7b,c^ ^]^ According to the literature, the structure of the amorphous carbon monolayers consists of nanocrystalline domains surrounded by continuous random network (CRN), the nanocrystalline domains are more conducting and CRN are less conducting.^[^ ^14^ ^]^ The high sheet resistance of amorphous carbon monolayers generated from DMMC_60_ indicates that there are a large number of defects and disorders in the structure, which is consistent with the TEM results.^[^ ^15^ ^]^

**Figure 4:**
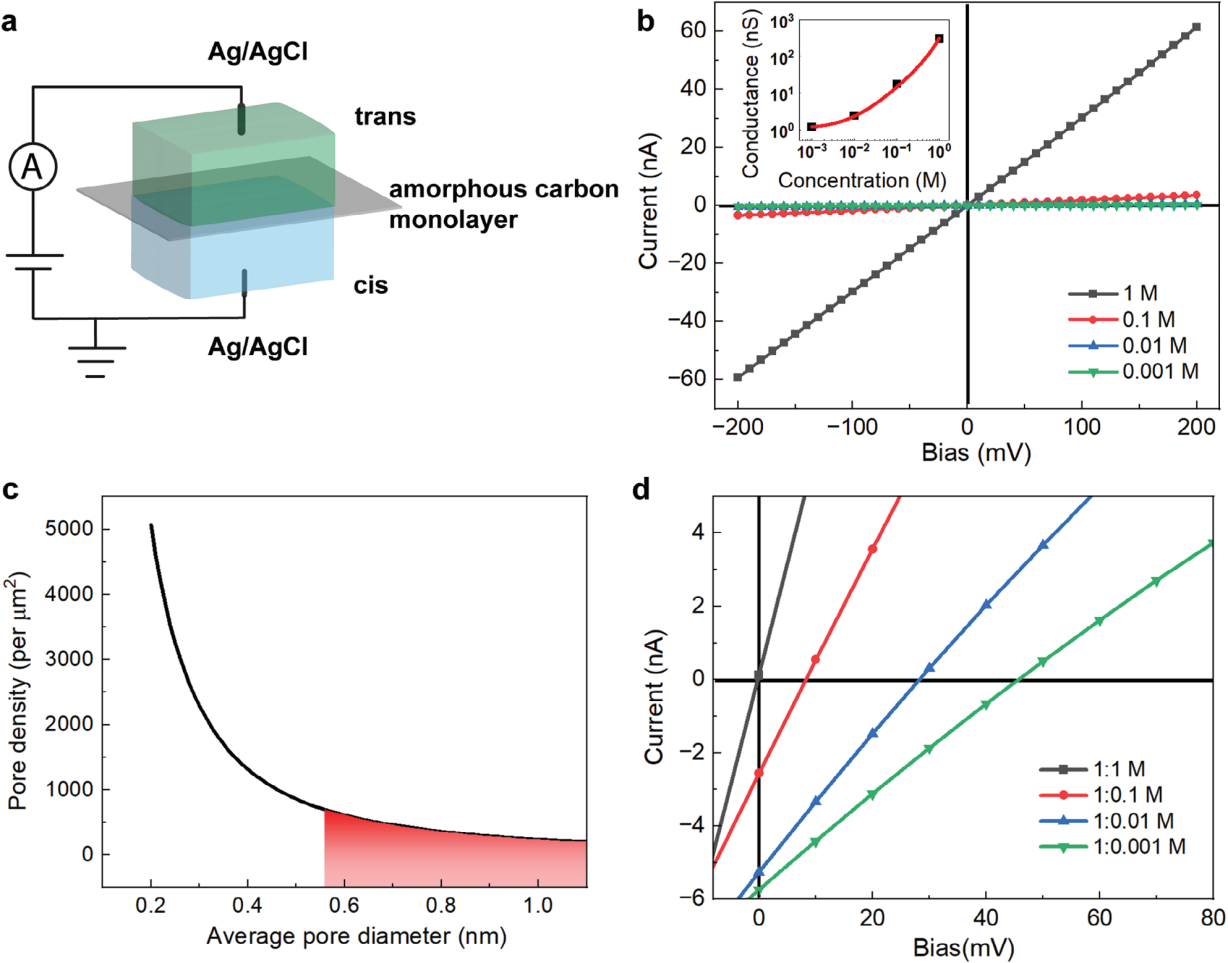
Ionic transmembrane transport measurements. a) Scheme illustrates the setup for ion transport measurement. b) The *I*–*V* curves of amorphous carbon membrane on support. Insert, conductivity versus salt solution concentration. c) The diagram of nanopore density versus average pore diameter of amorphous carbon monolayer, the pore density is predicted to be below 680 per µm^2^. d) The *I*–*V* curves of amorphous carbon membrane in KCl salt gradients.

Given the intense interest in semipermeable membranes, the ionic transmembrane transport behaviors of amorphous carbon monolayers were studied (see Experimental Section for details). The monolayer was transferred onto a glass support with a 1 µm diameter hole, and the support was placed between a homemade flow cell (**Figure** **4**a). KCl solution was selected as the K^+^ and Cl^−^ share similar bulk mobilities and negligible liquid junction potential. Ag/AgCl reference electrode was used to apply bias over the membrane and the current was recorded. The bare glass pore behaves as an ohmic resistor (Figure S5, Supporting Information). The resistance of amorphous carbon membrane on glass pores is significantly smaller than that of bare glass pores (Figure 4b). The *I*–*V* curves of amorphous carbon monolayer are linear, and no rectification effect was observed even for 1 mM KCl solution, indicating the absence of asymmetric charges on the membrane (Figure 4b; Figure S6, Supporting Information).^[^ ^4^, ^16^ ^]^ By decreasing the concentration of KCl solution, the conductance of the amorphous carbon membrane over a 1 µm hole decreases nonlinearly (Figure 4b, insert), indicating the existence of surface charge on the membrane.^[^ ^2^, ^7^ ^]^

The pore density of the amorphous carbon monolayer can be derived from Equation (1).^[^ ^4b^ ^]^ Where G is the conductance of the amorphous carbon membrane on glass support, *l* is the thickness of the amorphous carbon membrane, D is the average diameter of nanopore on amorphous carbon membrane, *σ* is the bulk solution conductivity (105 mS cm^−1^ for 1 M KCl). The resistance of the glass pore and amorphous carbon membrane on the glass pore is 0.46 and 3.25 MΩ, respectively. Thus, the conductance of the amorphous carbon membrane on 1 µm pore is ≈2.79 MΩ (358 nS). Considering that K^+^ and Cl^‐^ cannot transport through the nanopore with a diameter smaller than 0.6 nm efficiently,^[^ ^17^ ^]^ the pore density is below 680 per µm^2^.

(1) $$n=\frac{G\left(\frac{4l}{\pi D^{2}}+\frac{1}{D}\right)}{\sigma }$$

The ion selectivity of amorphous carbon membrane is also studied. KCl solution with concentrations ranging from 1 m to 1 mm is placed on *trans* side and 1 m KCl solution is placed on *cis* side of the flow cell. Under the concentration gradient, if the membrane is not ion‐selective, both anions and cations can migrate to the low‐concentration side at equal rates and no potential would arise. If the membrane is ion‐selective, one type of ion will diffuse through the membrane faster than the counter ion, generating a potential and a net current (without applying bias). The net current is negative, indicating that the amorphous carbon membrane is cation‐selective and negatively charged. The negative charge is usually generated from the protonatable groups, such as COO^−^, which comes from the oxidation of the nanopore edge and membrane.^[^ ^7^, ^18^ ^]^ The ion selectivity can be calculated from the open circuit potential E_oc_ (the potential corresponding to zero current) by using Equation (2).

(2) $$E_{oc}=S\frac{\mathrm{RT}}{\mathrm{zF}}ln\frac{\gamma_{h}c_{h}}{\gamma_{l}c_{l}}$$

where S is the membrane ion selectivity, R is the gas constant, T is the temperature, z is the ion valence, F is the Faraday constant, *γ* is the mean activity coefficient, and c is the KCl concentration. The ion selectivity of the membrane is about 26%, which is comparable to the ion selectivity of 2D materials reported in the literature.^[^ ^7^, ^19^ ^]^

## 3 Conclusion

The centimeter‐sized amorphous carbon monolayer was successfully synthesized directly on the Si wafer by pyrolyzing the monolayer of fullerene, a curved PAH. The amphiphilic nature of DMMC_60_ helps to form a monolayer. The amorphous carbon monolayer was fully characterized and the annealing process was studied in detail. The results show that the amorphous carbon monolayer fabricated with fullerene is metastable and has lower thermal stability than that prepared with planar PAHs. Electrical measurements show that the monolayer is insulating. The ionic transmembrane transport behavior was also studied and the amorphous carbon monolayer is cation‐selective with a selectivity of 26%. Our results provide new avenues for studying amorphous carbon monolayers and these special properties make it potentially useful in nanoelectronics,^[^ ^20^ ^]^ water desalination,^[^ ^21^ ^]^ and blue energy generation.^[^ ^22^ ^]^

## 4 Experimental Section

All chemicals were purchased commercially and used without further purification. The Langmuir–Blodgett film was prepared by using a KSV NIMA Langmuir trough from Biolin Scientific. Sample annealing was done with a rapid thermal processing (RTP) oven NBD1200 from Nobody Materials Science and Technology Co LTD. SEM images were recorded by Hitachi SU8230. Transmission electron microscope (TEM) images were recorded on FEI Talos F200s operated under 200 kV. Raman spectra were taken by using a InVia Qontor Raman system from Renishaw. XPS spectra were recorded by using ESCALAB Xi+ from ThermoFisher Scientific. The electrical conductivity and ion transmembrane transport were measured by using patch clamp axon 200B from Molecular Devices.

### Membrane Fabrication

Amphiphilic fullerene DMMC_60_ was synthesized following a literature procedure.^[^ ^23^ ^]^ The amorphous carbon membrane was prepared by rapid annealing of DMMC_60_ monolayer. The cleanness of the RTP oven was checked by annealing a piece of clean Si wafer at 1000 degrees for 2 mm. After cooling to room temperature, Raman spectroscopy was used to confirm that no carbon contamination was deposited on the Si wafer. DMMC_60_ monolayer was prepared with Langmuir–Blodgett trough from KSV NIMA and the surface pressure was measured with a Wilhelmy Pt plate. A DMMC_60_/chloroform solution (1 mg mL^−1^) was spread on the surface of the water in an LB trough. After 20 min, chloroform was fully evaporated and the barriers were slowly pushing DMMC_60_ together until the desired surface pressure was reached (10 mm m^−1^) at a speed of 1 mm min^−1^. The Si wafer with 300 nm SiO_2_ layer was cleaned with acetone and treated with air plasma (150 W, 1 min) to form a hydrophilic surface. The LB film was slowly transferred on the Si wafer at constant surface pressure (10 mN m^−1^) and a deposition speed of 1 mm min^−1^ by a Langmuir Schaefer method. The LB film on the Si wafer was then placed inside a RTP oven and annealed at 500 °C for 30 s at 1 bar pressure and Ar atmosphere, the heating speed was 30°C s^−1^ from 23–500 °C. After annealing, the oven cooled down naturally to room temperature, and the sample was taken out and used for characterization.

### PMMA Transfer

The PMMA transfer was carried out following a standard procedure. The silicon wafer was cut into pieces with a size of 5 mm × 5 mm, and a PMMA solution (950 K A4, Microchem) was spin‐coated onto the surface of the wafer at a speed of 4000 rpm. The thickness of the PMMA layer was ≈400 nm according to the spin‐coating curve provided by the supplier. The wafer with the PMMA layer on top was then placed on the surface of the HF/water solution (20%). After about 30 s, the SiO_2_ layer was etched and the wafer fell into the water, the PMMA layer + amorphous carbon monolayer was floated on the surface of the HF solution. The PMMA layer + amorphous carbon monolayer was taken out with a spoon and placed in clean water. This process was repeated three times to remove any contaminations. The PMMA layer + amorphous carbon monolayer was then transferred onto the desired substrates and the PMMA layer was removed with acetone.

### Electrical Conductivity Measurement

The amorphous carbon monolayer was transferred onto the interdigital electrode using the PMMA transfer method, and a patch clamp amplifier was used to measure the current and the resistance is calculated in the bias range of 0–200 mV.

### Ionic Transmembrane Transport Measurement

The amorphous carbon monolayer was transferred onto a glass plate with one 1 um diameter pore. The glass plate was placed in the middle of the PMMA flow cell. The flow cell was fully wet by treated with air plasma (60 W, 30 s) and later with a water/ethanol solution (5:5) before the measurement. The Ag/AgCl electrode was used to apply bias and it was prepared by placing the silver wire in a sodium hypochlorite solution for 10 min. Bias and current were recorded with a patch clamp.

## Conflict of Interest

The authors declare no conflict of interest.

## Author Contributions

M.H. and X.L. synthesized the modified fullerene molecule. M.H. performed the Langmuir‐Blodgett experiments, sample preparation, conductivity measurements, and ion transmembrane transport measurements. Y.D. performed AFM, SEM, and Raman studies. M.H. and X.L. wrote the manuscript and supervised the work.

## Supporting information

Supporting Information

## Data Availability

The data that support the findings of this study are available from the corresponding author upon reasonable request.
