# Supplementary material for: Nanoporous Amorphous Carbon Monolayer Derived from Fullerene Film
Source: Adv Sci (Weinh). 2023 Dec 28;11(10):2308187. doi: 10.1002/advs.202308187 (PMC10933613; doi:10.1002/advs.202308187)
Supplement: Supplementary file 1 — Supporting Information [file ADVS-11-2308187-s001.pdf]

ADVANCED SCIENCE
Open Access

# Supporting Information

for *Adv. Sci.*, DOI 10.1002/advs.202308187

Nanoporous Amorphous Carbon Monolayer Derived from Fullerene Film

*Meng He*, Yehui Ding and Xue Liu**

**Nanoporous amorphous carbon monolayer derived from fullerene film**

Meng He[1*], Yehui Ding[2], Xue Liu[2*]

1. College of New Energy, Xi'an Shiyou University, 710065 Xi'an, China
2. State Key Laboratory for Mechanical Behaviour of Materials, Xi'an Jiaotong University, 710049 Xi'an, China

E-mail: hemeng@xsyu.edu.cn; liuxue@xjtu.edu.cn

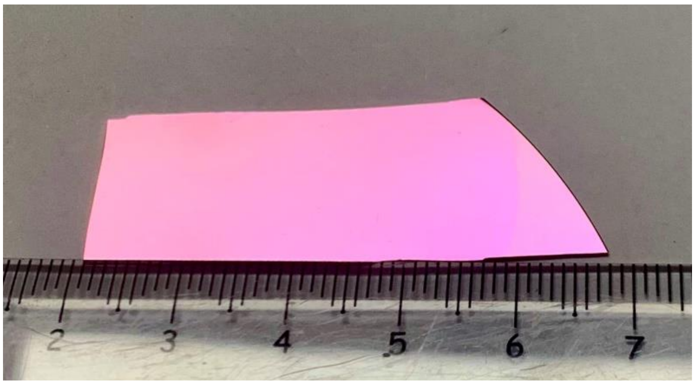


Supplementary Figure 1. The optical image of $DMMC_{60}$ monolayer on Si wafer before annealing.

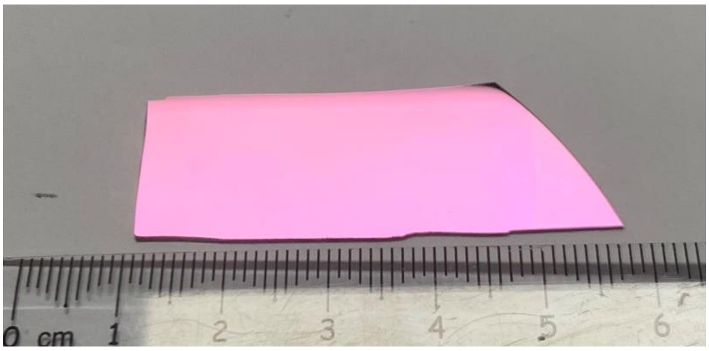


Supplementary Figure 2. The optical image of $DMMC_{60}$ monolayer on Si wafer after annealing.

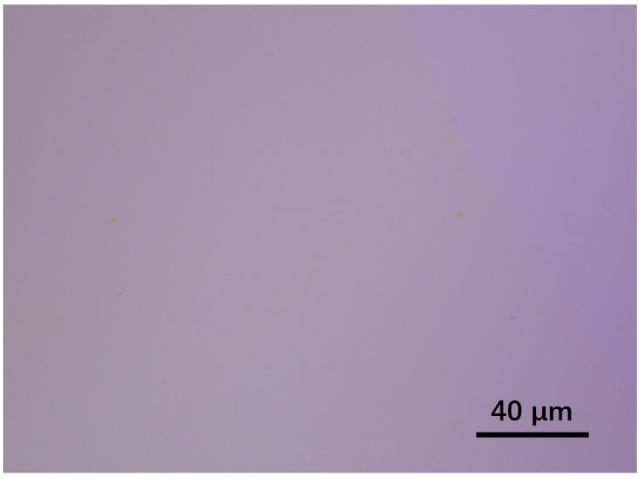


Supplementary Figure 3. The microscopic image of DMMC$_{60}$ monolayer on Si wafer after annealing

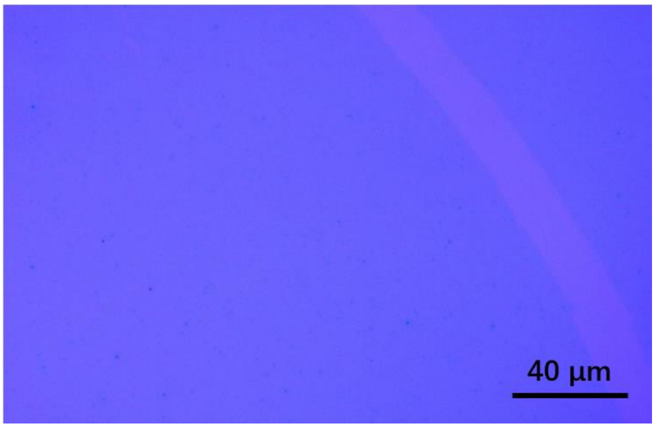


Supplementary Figure 4. The microscopic image of amorphous carbon monolayer on Si wafer prepared at 900 °C with planar PAHs, a crack can be observed on the right side. The details of its preparation will be reported in a separate work.

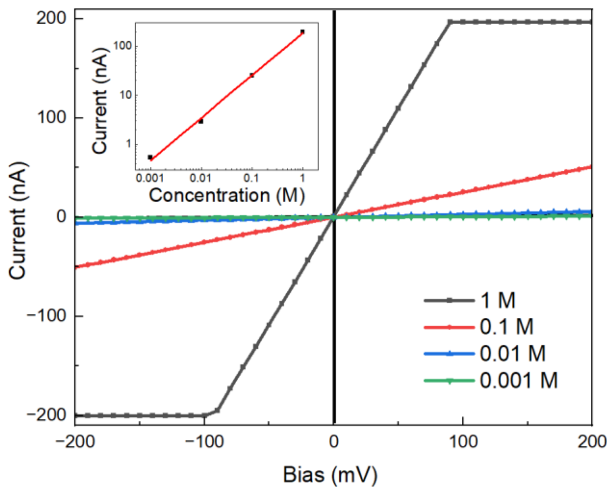

Supplementary Figure 5. The I-V curves of glass support with 1 μm hole. For 1 M KCl solution, the patch clamp is overload when the bias is larger than 90 mV.

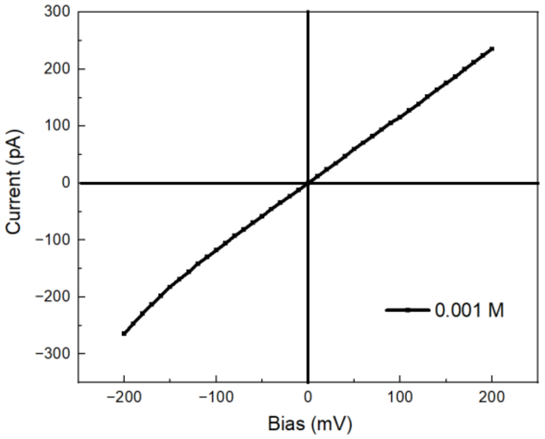


Supplementary Figure 6. The I-V curve of amorphous carbon membrane on glass pore in 1 mM KCl solution.
